# Verbal fluency dual-tasks show greater age-related cognitive-motor interference: a meta-analysis of walking performance

**DOI:** 10.1007/s00221-025-07169-7

**Published:** 2025-10-04

**Authors:** Kenneth Harrison, Keven Santa-Maria Guzman, Brandon M. Peoples, Silvia Campos-Vargas, Bria R. Smith, Damaris C. Cifuentes, Grace Greer, Kristina A. Neely, Jaimie A. Roper

**Affiliations:** Auburn University, Auburn, AL USA

**Keywords:** Gait speed, Serial subtraction, Executive function, Locomotion, Cerebellar function, Dual-task

## Abstract

A substantial body of literature has examined gait during cognitive dual-tasking in younger and older adults. However, it remains unclear how, and to what extent, different cognitive tasks uniquely influence gait. This meta-analysis quantified age-related differences in gait speed during dual-task walking. Importantly, we examined cognitive task as a potential moderator. We searched Web of Science for studies comparing young and older adults during single-task and dual-task walking conditions. Twenty-two studies met the inclusion criteria, representing 544 young adults (mean age range: 20–31 years) and 511 older adults (mean age range: 62–85 years). Studies employed primarily serial subtraction tasks (n = 12) and verbal fluency tasks (n = 8); however, one study used digit vigilance, and another used a texting paradigm during walking. Random-effects meta-analysis using standardized mean differences (Hedges' g) revealed a significant overall effect (g = −0.2612, 95% CI [−0.4914, –0.0310], *p* = 0.0261), indicating greater dual-task costs in older adults compared to younger adults with a small to medium effect size. Substantial heterogeneity was observed across studies (I^2^ = 66.53%, *p* < 0.0001). Subgroup analysis demonstrated that verbal fluency tasks produced a larger and statistically significant age-related difference (g = −0.4744, 95% CI [−0.8712, −0.0777], *p* = 0.0191), while serial subtraction tasks showed smaller, non-significant effects (g = −0.1412, *p* = 0.3474). These findings suggest that verbal fluency creates unique neural resource competition in older adults, involving prefrontal, cerebellar, and basal ganglia circuits that support both language production and gait control. The task-specific vulnerability to verbal fluency, and not serial subtraction, provides evidence for age-related changes in cognitive-motor integration. Rehabilitation strategies targeting executive functions may be effective for maintaining mobility in aging populations.

## Introduction

Successful human aging is characterized by the maintenance of mobility and cognitive function, and gait speed serves as a critical vital sign that predicts functional independence, quality of life, and survival in older adults (Fritz & Lusardi 2009). With advancing age, changes in neural, musculoskeletal, and cardiopulmonary systems can compromise the intricate coordination required for efficient locomotion. Dual-task walking—the simultaneous performance of walking and a secondary task—provides a sensitive paradigm for examining age-related changes in mobility (Beurskens & Bock 2012), because it challenges the integrated function of multiple physiological systems that undergo significant alterations across the lifespan (Beurskens et al. 2014). The neurobiology of healthy aging introduces susceptibilities to dual-task interference through several key mechanisms. First, age-related structural and functional changes in the cerebellum, basal ganglia, and prefrontal cortex reduce the neural efficiency of circuits that support both cognitive processing and motor control (Seidler et al. 2010). Second, white matter integrity declines with age, particularly in frontoparietal and frontocerebellar pathways critical for integrating sensorimotor information during complex tasks (Groh & Simons 2025). Evidence for these changes in neuroimaging studies revealed increased and more diffuse activation patterns in older adults during dual-task walking compared to younger counterparts (Fettrow et al. 2021), suggesting compensatory neural recruitment to maintain performance (Reuter-Lorenz & Cappell 2008). This increased neural resource utilization likely reflects diminished processing capacity and reduced automaticity of both cognitive and motor functions with advancing age (Richardson et al. 2024; Seidler et al. 2010).

The age-related changes in dual-task performance can be understood within the broader theoretical framework of neural compensation in aging. The Compensation-Related Utilization of Neural Circuits Hypothesis (CRUNCH) posits that older adults compensate for declining neural efficiency by recruiting additional brain regions, particularly at lower levels of task difficulty (Gerver et al. 2020; Reuter-Lorenz & Cappell 2008). Similarly, the Posterior-to-Anterior Shift in Aging (PASA) theory suggests that older adults show increased frontal activation compared to younger adults as a compensatory mechanism to maintain cognitive performance (Davis et al. 2008). Therefore, we suggest that during dual-task walking, these compensatory recruitment patterns may become insufficient when cognitive demands exceed available neural resources, leading to the pronounced performance decrements observed in older adults. We suggest that the greater dual-task costs in aging reflect not just peripheral changes in muscle function or joint mechanics, but fundamental alterations in how the brain allocates and coordinates neural resources across cognitive and motor domains.

From a biomechanical perspective, aging brings about significant changes in lower extremity function that become particularly evident during dual-task walking. Older adults typically exhibit a distal-to-proximal redistribution of mechanical work, relying less on the ankle and more on the hip for propulsion and power generation during walking (Franz 2016). This altered joint kinetic profile may represent a central nervous system adaptation to age-related peripheral changes, such as reduced ability of the calf muscle and Achilles tendon to store and return energy, manifesting as reductions in ankle propulsion and gait speed (Boyer et al. 2023). During dual-tasking these gait insufficiencies become more pronounced, and we hypothesize that the addition of cognitive load imposed by the secondary task is one of the major influences of the ankle-hip kinetic relationship. Furthermore, older adults show greater stride-to-stride variability under dual-task conditions (Nedović et al. 2024) which is associated with increased fall risk and may reflect diminished capacity for online error correction with advancing age (Hollman et al. 2007). This increased variability likely stems from the competing demands for neural resources during dual-tasking, where older adults must continuously reallocate attention between cognitive processing and the moment-to-moment adjustments required for consistent gait patterns (Hollman et al. 2007).

The theoretical underpinning of age-related dual-task interference has been previously explained through attentional capacity and resource competition models (Wickens 2008). Specifically, the Multiple Resource Theory posits that performance decrements during dual tasking are related to shared neural resources (Wickens 2008) and these decrements become more pronounced with age as overall processing resources diminish (Nóbrega-Sousa et al. 2020). This theory is complimented by research that identified specific neural signatures of age-related dual-task performance Chmielewski et al. 2014), demonstrated the role of cognitive reserve in moderating dual-task costs in older adults (Yordanova et al. 2021), and established new paradigms for assessing age-related changes in task prioritization (Al-Yahya et al. 2011). Furthermore, evidence suggests that different types of cognitive tasks (e.g., working memory versus executive function) may have distinct effects on gait speed across different age groups (Amboni et al. 2013; Kearney et al. 2013; Watson et al. 2010).

A seminal meta-analysis by Smith and colleagues (2016) established that cognitive dual-tasks significantly reduce gait speed in healthy older adults, with the magnitude of interference varying by cognitive task type (Smith et al. 2016). Mental tracking tasks—such as serial subtraction, which engage working memory and sustained attention through continuous numerical processing—produced average gait speed reductions of 0.19 m/s. The minimal clinically important difference for gait speed is shown to be around 0.1–0.2 m/s (Bohannon & Glenney 2014). In contrast, verbal fluency tasks—which require language production, semantic memory access, and executive control for generating words within categories or starting with specific letters—resulted in smaller but still clinically significant reductions of 0.13 m/s. These differential effects reflect the distinct neural circuits recruited by each task type. Mental tracking tasks primarily activate bilateral frontoparietal working memory networks and dorsolateral prefrontal cortex (Wager & Smith 2003), while verbal fluency tasks engage left-lateralized language production areas including Broca's area, left inferior frontal gyrus, and temporal-parietal language networks (Costafreda et al. 2006). The varying degrees of overlap between these cognitive networks and the neural circuits supporting gait control may explain the task-specific patterns of dual-task interference observed across different cognitive demands.

Recent studies have demonstrated that the magnitude of dual-task interference when performing these various cognitive tasks are a valuable predictor of falls (Tsang et al. 2022) and cognitive decline (Ramírez & Gutiérrez 2021; Yang et al. 2020) in aging populations. However, no systematic quantitative synthesis has directly compared dual-task effects between young and older adults or between cognitive task type to isolate the specific contribution of aging to cognitive-motor interference. Therefore, the purpose of this meta-analysis was to quantitatively synthesize the available evidence comparing dual-task effects on gait speed between young and older adults. Building upon previous findings from Smith and colleagues (2016), we aimed to: (1) determine the magnitude of age-related differences in cognitive-motor dual-task costs on gait speed and (2) examine potential moderating factors such as the type of secondary task and task complexity. Understanding these age-related differences is crucial for developing targeted interventions that address the specific mechanisms of mobility decline in the aging population.

## Methods

### Search strategy and study selection

This systematic review and meta-analysis followed the Preferred Reporting Items for Systematic Reviews and Meta-Analyses (PRISMA) guidelines (Page et al. 2021). We conducted a comprehensive search of the Web of Science database using the following search terms: ("older adult*" OR elder* OR aging OR aged OR geriatric* OR senior*) AND ("young adult*" OR young OR youth*) AND ("dual task*" OR "dual-task*" OR "cognitive task*" OR "secondary task*" OR "concurrent task*" OR "divided attention") AND ("gait" OR "gait speed*" OR "walking speed*" OR "gait velocity" OR "walking velocity" OR "spatiotemporal parameter*"). No date restrictions were applied to maximize the identification of relevant studies. Three researchers (KH, SV, GG) independently screened titles and abstracts of identified studies. Disagreements were resolved through consensus discussion between the same group. Full-text articles were retrieved for studies that potentially met the inclusion criteria, and these were subsequently assessed for eligibility by the same three researchers.

### Inclusion and exclusion criteria

Studies were included if they met the following criteria: (1) included both young and older adult groups, with age classifications following National Institutes of Health guidelines (*Age | National Institutes of Health (NIH)*, 2025); (2) measured gait speed during both single-task and dual-task walking conditions; (3) reported means and standard deviations for gait speed in both conditions for both age groups; (4) employed standard walking protocols (e.g., walking on a level surface at self-selected pace); and (5) used dual-task paradigms that aligned with preset protocol paradigms in the literature such as verbal fluency and working memory or serial seven subtraction. Studies were excluded if they: (1) examined incorrect settings such as modified walking under a certain constraint or environment (over obstacles) (n = 4); (2) measured outcomes other than gait speed (n = 34); (3) used interventions that did not align with standard dual-task protocols (n = 2); (4) employed inappropriate study designs such as being queued to prioritize a certain task, or using a modified unique dual task protocol (n = 54); or (5) included patient populations with specific pathologies rather than healthy adults (n = 12).

### Data extraction

From each included study, we extracted the following information: (1) study characteristics (author names, publication year); (2) participant demographics (sample sizes for young and older adult groups); (3) dual-task characteristics (type of secondary cognitive task); (4) gait parameters (mean and standard deviation of gait speed for both single-task and dual-task conditions in both age groups). The extracted data were organized according to the cognitive task employed, which we categorized into two groups: serial subtraction tasks (Task 1), and verbal fluency tasks (Task 2). This classification was based on the distinct cognitive processes engaged by each task type and informed by previous research on dual-task taxonomy.

### Statistical analysis

*Effect Size Calculation* Standardized mean differences (SMDs) were calculated to quantify the magnitude of age-related differences in dual-task costs on gait speed. For each study, we computed the difference between young and older adults in their respective dual-task effects (DTE), defined as the percentage change in gait speed from single-task to dual-task conditions. Negative effect sizes indicate greater dual-task costs in older adults compared to younger adults. The standardized mean difference was calculated using Hedges' g to correct for small sample bias, with the following formula: where *mean₁* and *mean₂* represent the mean dual-task effect for older and younger adults, respectively; *sd₁* and *sd₂* represent the standard deviations; and *n₁* and *n₂* represent the sample sizes.

*Meta-Analytic Procedures* We conducted a random-effects meta-analysis using the restricted maximum likelihood (REML) estimator, which is optimal for estimating between-study variance in meta-analyses with heterogeneous effect sizes. The random-effects model was selected a priori due to the expected heterogeneity across studies resulting from variations in dual-task paradigms, participant characteristics, and methodological approaches. Data extracted from each study was stored in Microsoft Excel for Microsoft 365 MSO (Version 2403 Build 16.0.17425.20176) 64-bit. All statistical analyses and visualizations in this meta-analysis were conducted in R studio (Posit Software, version 2023.12.0, PBC, Build 369) using R (version 4.3.1 and the metafor package (version 4.6.0) (Viechtbauer 2010). Between-study heterogeneity was quantified in R using τ^2^ (estimated amount of total heterogeneity), I^2^ (percentage of total variability due to heterogeneity), and Cochran's Q test. To explore potential moderators of the observed effects, we conducted subgroup analyses by cognitive task type. Studies were categorized into two groups based on the secondary cognitive task employed: serial subtraction tasks (Task 1) and verbal fluency tasks (Task 2). A meta-regression using the Knapp-Hartung adjustment method was subsequently performed to evaluate the moderating effect of task type on the magnitude of age-related differences in dual-task costs.

*Publication Bias and Sensitivity Analyses* Potential publication bias was assessed through visual inspection of funnel plots (Fig. 3) and formal statistical tests, including both the meta-regression test and the weighted regression test for funnel plot asymmetry. A profile likelihood plot was generated to evaluate the stability of the τ^2^ estimate, and a Baujat plot was used to identify potentially influential studies. A residual funnel plot was examined to assess whether residual heterogeneity showed systematic patterns after accounting for task type as a moderator. Case wise diagnostics, including standardized residuals, Cook's distance, and covariance ratios, were computed to identify studies with disproportionate influence on the overall results.

## Results

### Study selection

**Fig. 1:**
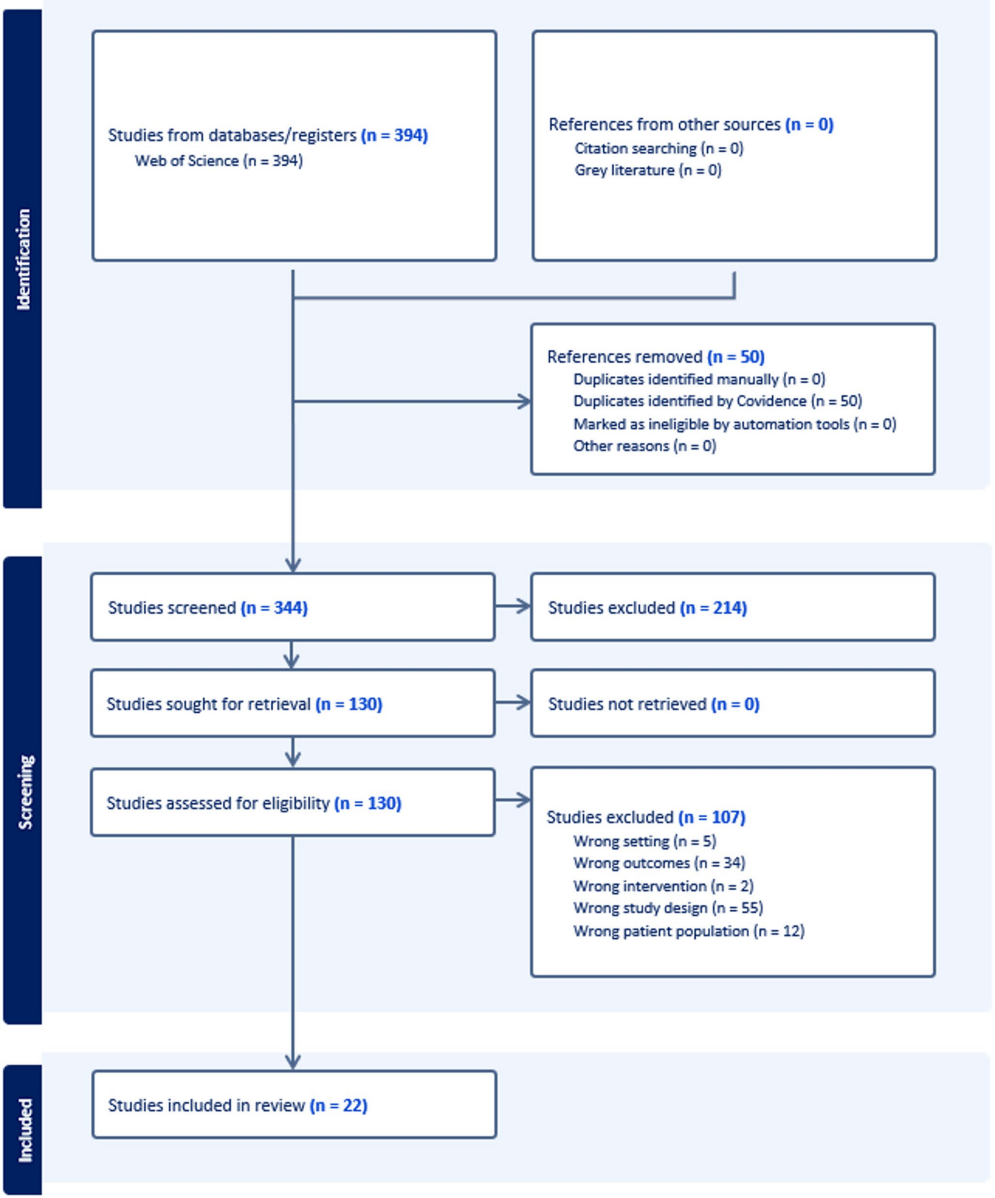
PRISMA Flow Diagram PRISMA flow diagram showing the systematic search and study selection process. The initial database search yielded 394 records from Web of Science. After removing duplicates and applying inclusion/exclusion criteria, 22 studies were included in the final quantitative synthesis

The initial database search yielded 394 records from Web of Science. After removing fifty duplicates identified using Covidence, 344 studies remained for title and abstract screening. Of these, 214 were excluded as they did not meet the initial screening criteria. The remaining 130 full-text articles were assessed for eligibility, resulting in the exclusion of 107 studies: 5 examined incorrect settings, 34 measured outcomes other than gait speed, 2 used interventions that did not align with standard dual-task protocols, 55 employed inappropriate study designs, and 12 included patient populations with specific pathologies. Thus, twenty-two studies were included in the final quantitative synthesis (Fig. 1).

### Characteristics of included studies

The twenty-two included studies were published between 2007 and 2023, representing a diverse temporal range of research on dual-task effects. Sample sizes ranged from 14 to 43 participants per age group across studies. The mean age for younger adult groups ranged from 20 to 31 years, whereas older adult groups ranged from 62 to 85 years (Table 1). Studies employed various cognitive tasks during dual-task walking, which we generally categorized into two types: serial subtraction tasks (Task 1, n = 12 studies), verbal fluency tasks (Task 2, n = 8 studies). There were two studies that met inclusion criteria while using slightly different tasks such as digit vigilance and texting while walking. Serial subtraction tasks primarily involved counting backward by specified intervals (e.g., 3 s, 7 s). Verbal fluency tasks included naming items within specified categories or generating words beginning with designated letters. All included studies measured gait speed during both single-task walking and while performing concurrent cognitive tasks. The dual-task assessment protocols were conducted in laboratory settings with participants walking on level surfaces, although the specific walking distances varied across studies.

**Table 1:** Summary of included studies. Age is in years ± SD

Author	Year	Young Adult Sample Size	Old Adult Sample Size	Young Adult Mean Age	Old Adult Mean Age	Cognitive Dual-Task
Agner et al	2015	16	16	23 ± 2	85 ± 1	Serial Subtraction
Asai et al	2019	28	43	23 ± 5	74 ± 6	Serial Subtraction
Baek et al	2023	20	20	30 ± 3	64 ± 3	Serial Subtraction
Granacher et al	2010	16	16	24 ± 1	72 ± 5	Serial Subtraction
Granacher et al	2010	18	18	22 ± 3	74 ± 6	Serial Subtraction
Hassan et al	2022	20	17	28 ± 3	71 ± 6	Verbal Fluency
Hawkins et al	2018	9	15	22 ± 3	78 ± 5	Verbal Fluency
Hoang et al	2022	25	25	24 ± 5	62 ± 4	Serial Subtraction
Hollman et al	2007	20	20	25 ± 3	81 ± 5	Verbal Fluency
Hseih et al	2012	15	15	26 ± 2	73 ± 5	Verbal Fluency
Hupfield et al	2022	37	23	21 ± 2	73 ± 10	Serial Subtraction
Kaewkaen et al	2021	32	32	20 ± 1	68 ± 3	Serial Subtraction
Klotzbier et al	2022	14	12	28 ± 6	72 ± 8	Verbal Fluency
Krasovsky et al	2018	30	20	28 ± 4	69 ± 4	Texting While Walking
Mirelman et al	2017	23	20	31 ± 4	70 ± 6	Serial Subtraction
Nobrega-Sousa et al	2020	15	15	22 ± 1	71 ± 1	Digit Vigilance
Piche et al	2023	24	26	23 ± 3	69 ± 4	Verbal Fluency
Priest et al	2008	19	23	23 ± 2	80 ± 9	Serial Subtraction
Prupetkaew et al	2019	12	12	23 ± 2	73 ± 5	Verbal Fluency
Soangra et al	2017	7	7	22 ± 2	71 ± 6	Serial Subtraction
Soma et al	2010	30	30	26 ± 3	69 ± 3	Serial Subtraction
Yogev-Seligmann et al	2010	40	17	27 ± 2	72 ± 7	Verbal Fluency

The basal ganglia help sequence motor actions and cognitive operations (Leisman & Melillo 2013; Middleton & Strick 2000). This subcortical system, which includes the striatum (caudate nucleus and putamen), substantia nigra, and globus pallidus, relies heavily on dopaminergic signaling for proper function. The striatum, as the primary input nucleus of the basal ganglia, receives extensive dopaminergic innervation from the substantia nigra that is essential for both motor sequencing and cognitive control processes. Age-related changes in striatal dopamine function—including reduced dopamine receptor density, decreased dopamine synthesis, and altered dopamine transporter availability (Volkow et al. 1998) —affect the efficiency of motor and cognitive control (Bäckman et al. 2006; Volkow et al. 1998). When verbal fluency and walking occur simultaneously, the basal ganglia must support two sequencing tasks at once, creating processing conflicts (Seidler et al. 2010; Yu et al. 2007) that manifest primarily in reduced gait speed. Dual-sequencing demand is particularly challenging for older adults who show reduced automaticity in both cognitive and motor sequencing (Hassan et al. 2022; Hausdorff et al. 2008). The continuous nature of verbal fluency may create an ongoing, unpredictable cognitive load that makes it difficult to establish a stable rhythm of resource allocation between cognitive processing and motor control monitoring. Our finding that verbal fluency tasks produce significantly greater age-related dual-task costs—compared to serial subtraction tasks—may reflect this distinct sequencing challenge on the basal ganglia structures, as serial subtraction follows a more predictable pattern.

### Overall effect

**Fig. 2:**
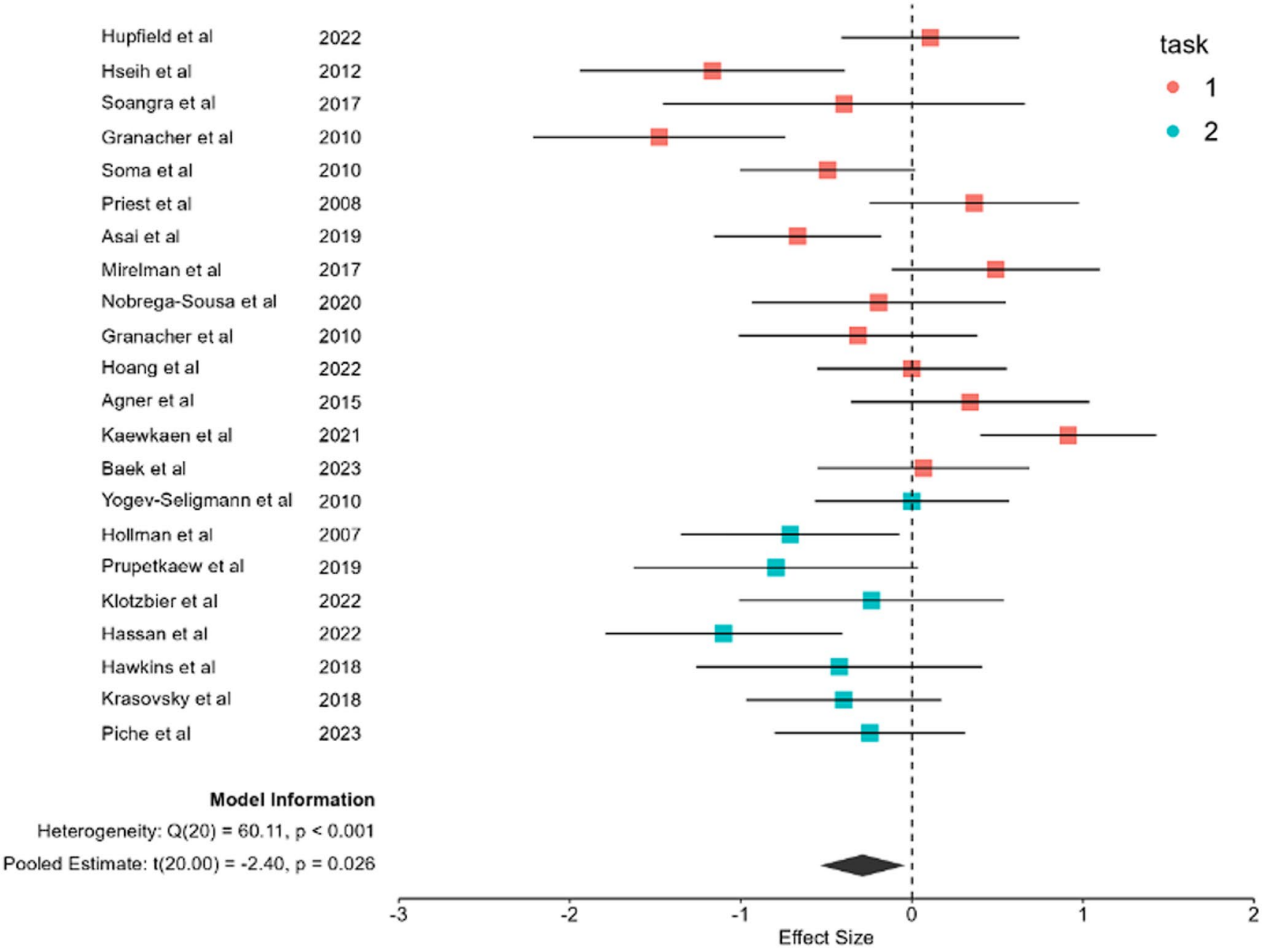
Forest Plot of Age-Related Dual-Task Effects on Gait Speed Forest plot displaying standardized mean differences (Hedges' g) and 95% confidence intervals for individual studies comparing dual-task costs between young and older adults. Effect sizes are color-coded by cognitive task type: red identifies serial subtraction and green identifies verbal fluency. Negative effect sizes indicate greater dual-task costs in older adults. The diamond denotes the overall pooled effect size

The random-effects meta-analysis of 22 studies revealed a significant overall effect size of g = −0.2612 (95% CI [−0.4914, −0.0310], *p* = 0.0261), indicating that older adults experience significantly greater dual-task costs in gait speed compared to younger adults (Fig. 2). The analysis revealed substantial heterogeneity across studies (τ^2^ = 0.2055, I^2^ = 66.53%, Q(22) = 65.80, *p* < 0.0001), suggesting considerable variability in the magnitude of age-related differences in dual-task costs. Figure 2 presents the forest plot of all included studies, color-coded by task type (Task 1: red; Task 2: green). This visualization highlights the pattern of more consistently negative effects for verbal fluency tasks and more variable effects for serial subtraction tasks, despite the formal moderation test not reaching statistical significance.

### Moderator analysis

The subgroup analysis by cognitive task type demonstrated that the magnitude of age-related differences in dual-task costs varied by the nature of the concurrent cognitive task. Task 2 (verbal fluency) showed the largest and only statistically significant effect (g = −0.4744, 95% CI [−0.8712, -0.0777], *p* = 0.0191), indicating greater dual-task costs among older adults compared to younger adults during verbal fluency tasks. Task 1 (serial subtraction) showed a smaller, non-significant effect (g = −0.1412, 95% CI [−0.4358, 0.1534], *p* = 0.3474). A meta-regression using the Knapp-Hartung adjustment method was conducted to further examine task type as a moderator. The omnibus test for moderation did not reach statistical significance (QM(df = 2) = 6.67, *p* = 0.0832), suggesting that task type may explain some of the heterogeneity in effects. The pattern of coefficients was consistent with the subgroup analysis, with Task 2 showing a significant negative effect. After accounting for task type, the residual heterogeneity remained significant (QE(20) = 60.11, *p* < 0.001), though the proportion of variance explained by heterogeneity remained substantial (I^2^ = 66.59%).

### Publication bias and sensitivity analysis

**Fig. 3:**
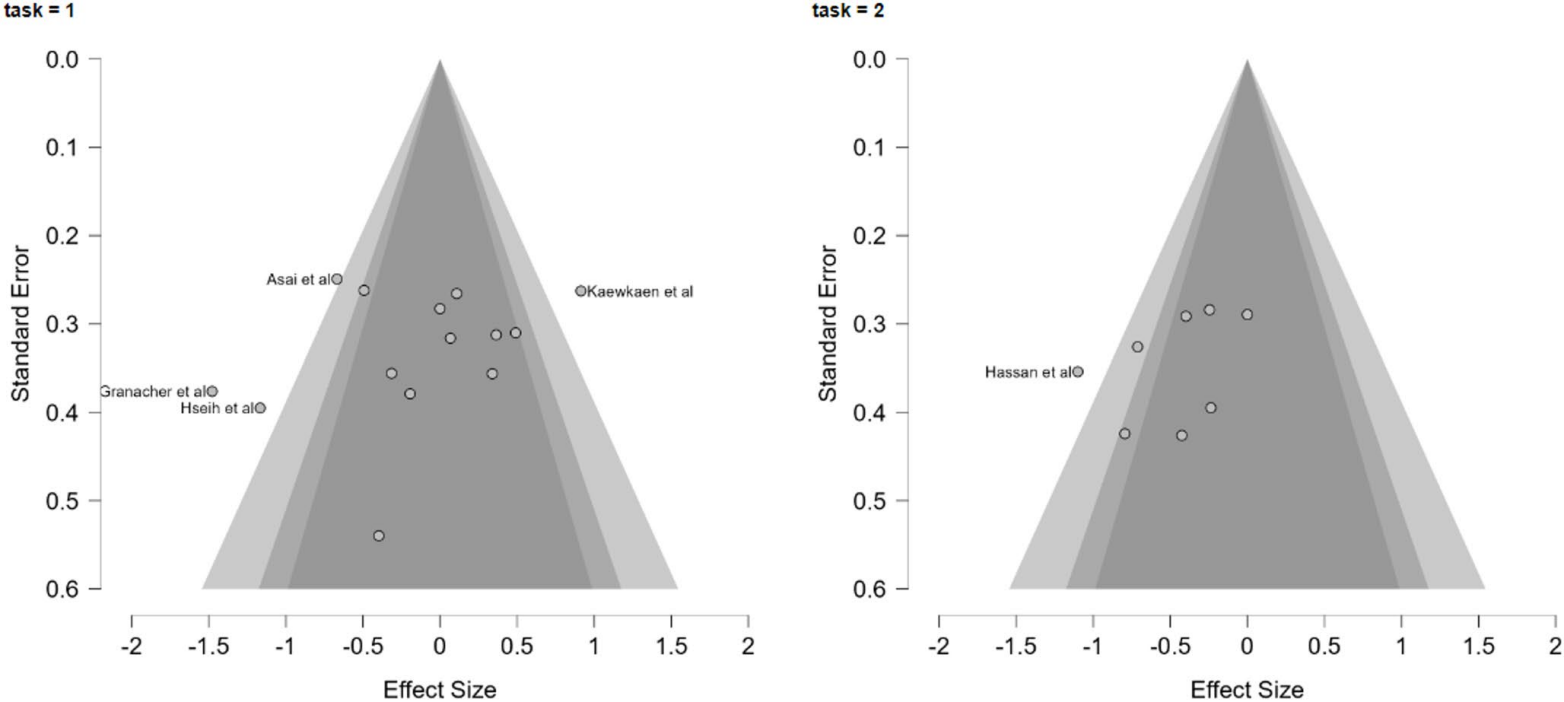
Funnel Plot for Assessment of Publication Bias Funnel plot examining potential publication bias across included studies. The x-axis shows standardized mean differences (Hedges' g) and the y-axis shows standard error. The vertical line represents the overall effect size, and the diagonal lines represent the expected 95% confidence interval bounds. Symmetrical distribution of studies around the effect estimate suggests minimal publication bias

Visual inspection of the funnel plot revealed a symmetric distribution of studies, suggesting limited evidence of publication bias (Fig. 3). This was confirmed by non-significant results on both the meta-regression test for funnel plot asymmetry (z = −1.298, *p* = 0.194) and the weighted regression test (t = −1.146, df = 22, *p* = 0.264). The profile likelihood plot for τ^2^ showed a stable estimate of between-study variance, with the maximum likelihood occurring at approximately τ^2^ = 0.28. The residual funnel plot showed no obvious asymmetry or patterning of residuals after accounting for task type as a moderator, suggesting that the meta-regression model appropriately captured the relationship between task type and effect size, though substantial unexplained heterogeneity remained.

## Discussion

This meta-analysis quantified age-related differences in dual-task effects on gait speed. Further, we examined potential moderating factors across two different types of cognitive tasks. We have two novel findings. First, our meta-analysis showed a significant overall effect, indicating that older adults experience greater dual-task costs in gait speed compared to younger adults. Secondly, our subgroup analysis demonstrated that the nature of the cognitive task significantly moderates this age-related difference, with verbal fluency tasks producing the most pronounced and only statistically significant effect. These findings align with our hypothesis that task type influences the magnitude of age-related differences in dual-task performance. Our results suggest that the specific cognitive processes engaged by verbal fluency create unique challenges for older adults while walking compared to younger adults.

The substantial heterogeneities observed across studies (I^2^ = 66.53%) highlight the complexity of factors influencing dual-task performance across the lifespan. Although our meta-regression analysis did not reach levels of conventional significance (*p* = 0.0832), the pattern of results supports the conclusion that gait-speed alterations during walking while talking are particularly sensitive to age-related changes in cognitive-motor integration, especially when compared to serial subtraction. The current work replicates and extends the previous meta-analysis from Smith et al. (2016), which documented significant gait speed reductions during dual-tasking in older adults but did not directly compare age groups or examine task-specific effects. Several interconnected neurophysiological mechanisms, discussed below, may explain why dual-task walking is sensitive to performance decrements during verbal fluency tasks. We explore these mechanisms and their implications for understanding age-related changes in mobility.

### Neural resource competition

Verbal fluency tasks place substantial demands on frontal executive functions, particularly those mediated by structures like the dorsolateral prefrontal cortex (DLPFC) and anterior cingulate cortex (ACC) (Panikratova et al. 2020). Importantly, frontal regions become increasingly recruited for gait control in older adults compared to younger counterparts (Belli et al. 2021; Mirelman et al. 2017; Nóbrega-Sousa et al. 2020), possibly as a compensatory neural mechanism to maintain performance during age-related declines in motor control (Park & Reuter-Lorenz 2009; Rosano et al. 2012). When older adults coordinate verbal fluency with walking, they experience a neural resource bottleneck, where limited neural resources must be divided between cognitive and motor demands (Yogev-Seligmann et al. 2008). Neuroimaging studies by Holtzer et al. (2011) using functional near-infrared spectroscopy (fNIRS) have demonstrated increased prefrontal activation during verbal fluency dual-task walking compared to single-task walking, with overactivation being more pronounced in older adults (Holtzer et al. 2011). Consistent with Holtzer’s suggestion, the current analysis of behavioral studies demonstrates significant age-related differences to verbal fluency dual-task cost, with older adults showing more pronounced reductions in gait speed.

### Cerebellar involvement in speech and gait coordination

The cerebellum plays a critical role in the precise timing of gait cycles and the articulatory planning for speech production (Ackermann 2008; Schmahmann 2019). This dual involvement makes cerebellar function particularly relevant to understanding age-related differences in verbal fluency dual-task performance. Neuroimaging research by Bernard & Seidler (Bernard & Seidler 2014) has demonstrated that cerebellar volume decreases with age and that resting-state functional connectivity between the cerebellum and prefrontal regions becomes less efficient (Bernard & Seidler 2013; Rosano et al. 2007; Taniwaki et al. 2007). These age-related changes compromise the cerebellum's capacity to coordinate multiple functions simultaneously, creating a processing bottleneck during dual-task conditions.

The cerebellum's contribution to verbal fluency extends beyond motor speech control to include verbal working memory and word-finding processes essential for successful task performance (Marvel & Desmond 2010). During verbal fluency dual-task walking, the cerebellum must coordinate articulatory planning, maintain temporal sequencing of speech output, and simultaneously support the rhythmic timing of gait cycles (Bodranghien et al. 2016). This competing demand for cerebellar resources becomes particularly problematic in older adults with diminished cerebellar capacity (Taniwaki et al. 2007), manifesting as the pronounced gait speed reductions observed in our meta-analysis. The task-specific sensitivity to verbal fluency, rather than serial subtraction, may reflect the cerebellum's specialized role in language production timing, which creates unique resource competition not present during purely numerical cognitive tasks.

### Basal ganglia function and motor sequencing

The basal ganglia help sequence motor actions and cognitive operations (Leisman & Melillo 2013; Middleton & Strick 2000). This subcortical system, which includes the striatum (caudate nucleus and putamen), substantia nigra, and globus pallidus, relies heavily on dopaminergic signaling for proper function. The striatum, as the primary input nucleus of the basal ganglia, receives extensive dopaminergic innervation from the substantia nigra that is essential for both motor sequencing and cognitive control processes. Age-related changes in striatal dopamine function—including reduced dopamine receptor density, decreased dopamine synthesis, and altered dopamine transporter availability (Volkow et al. 1998) —affect the efficiency of motor and cognitive control (Bäckman et al. 2006; Volkow et al. 1998). When verbal fluency and walking occur simultaneously, the basal ganglia must support two sequencing tasks at once, creating processing conflicts (Seidler et al. 2010; Yu et al. 2007) that manifest primarily in reduced gait speed. Dual-sequencing demand is particularly challenging for older adults who show reduced automaticity in both cognitive and motor sequencing (Hassan et al. 2022; Hausdorff et al. 2008). The continuous nature of verbal fluency may create an ongoing, unpredictable cognitive load that makes it difficult to establish a stable rhythm of resource allocation between cognitive processing and motor control monitoring. Our finding that verbal fluency tasks produce significantly greater age-related dual-task costs—compared to serial subtraction tasks—may reflect this distinct sequencing challenge on the basal ganglia structures, as serial subtraction follows a more predictable pattern.

### Attentional demands and task prioritization

In addition to these neural mechanisms, our results align with motor control perspectives that suggest verbal fluency may have the capacity to disrupt the attentional demands of gait more severely than other cognitive tasks because it creates unpredictable attentional shifts (Shao et al. 2014). During verbal fluency tasks, older adults experience moments of increased cognitive load where they are tasked with holding the exclusion rule in mind, generating target words, and scanning words for accuracy (Stolwyk et al. 2015). This variable attentional rhythm might interfere with the steady, rhythmic attentional demands of walking, leading to inconsistent gait parameters that ultimately reduce overall speed. According to the "posture first" hypothesis proposed by Shumway-Cook et al. (1997), when facing competing demands, older adults typically prioritize postural stability over cognitive performance as a protective mechanism against falls (Shumway-Cook et al. 1997). Our results show verbal fluency, being an open-ended, generative task without clear completion criteria, may be particularly difficult to strategically deprioritize during dual-tasking, leading to more pronounced gait interference than tasks with discrete elements like serial subtraction.

## Limitations and future directions

Several limitations of our meta-analysis should be acknowledged. First, despite our comprehensive search strategy, the small number of studies for Task 2 (verbal fluency, n = 8) limited statistical power for subgroup comparisons. Second, the studies employed various gait assessment protocols, contributing to the substantial heterogeneity observed. Third, most studies focused exclusively on gait speed, preventing the investigation of other important gait parameters that reveal additional aspects of age-related differences in dual-task performance.

The finding that verbal fluency tasks create the more pronounced age-related differences in dual-task walking performance compared to serial subtraction reveals important insights about the neural mechanisms underlying age-related changes in mobility. The sensitivity of gait speed to verbal fluency suggests that certain executive functions—particularly those involving response generation, inhibition, and working memory, are especially relevant to understanding age-related changes in gait control. Future research should directly measure joint kinetics during verbal fluency dual-task walking to quantify the distribution of mechanical work across lower limb joints. This would move beyond kinematic observations that dual-tasking affects gait speed and toward a mechanistic understanding of how cognitive load reshapes the kinetic strategy for locomotion in older adults. In addition, multimodal studies, which integrate simultaneous neurophysiological recordings with motion capture, are necessary to quantify how cognitive load impacts gait.

## Conclusion

This meta-analysis provides robust evidence that older adults experience significantly greater dual-task costs in gait speed compared to younger adults, with verbal fluency tasks revealing more pronounced age-related differences than mental tracking tasks. These findings highlight the complex interplay between cognitive and motor processes across the lifespan and suggest that verbal fluency paradigms offer a unique approach for detecting age-related changes in gait control. The task-specific vulnerability to verbal fluency interference supports a multifaceted neurophysiological model involving prefrontal resource competition, cerebellar coordination challenges, and basal ganglia sequencing demands. Beyond their theoretical significance, these results have direct clinical implications, suggesting that rehabilitation strategies targeting the specific executive functions engaged during verbal fluency may be particularly effective for improving gait in aging populations. Moreover, our findings challenge previous explanations for age-related gait changes being related to peripheral nervous system changes only, instead supporting an integrated cognitive-motor control framework where the well-documented biomechanical adaptations in older adults' gait are meaningfully influenced by central neural processes. Future research, combining dual-task paradigms with detailed biomechanical analyses, is essential for fully characterizing how cognitive load reshapes locomotor strategies across the lifespan. Such research is paramount for the development of targeted interventions to maintain mobility and reduce fall risk in aging populations.

## Data Availability

The datasets generated and analyzed during the current study are available from the corresponding author upon reasonable request.
